# Patientengruppenspezifische Datenhoheitsbedürfnisse und Akzeptanz der elektronischen Patientenakte

**DOI:** 10.1007/s00103-022-03589-w

**Published:** 2022-09-23

**Authors:** Marc Baron von Osthoff, Ute Watzlaw-Schmidt, Thomas Lehmann, Jutta Hübner

**Affiliations:** 1 Fakultät Medien, Hochschule Mittweida, Technikumplatz 17, 09648 Mittweida, Deutschland; 2 Palliativstützpunkt Hameln-Pyrmont, Hameln, Deutschland; 3 Institut für Medizinische Statistik, Informatik und Datenwissenschaften, Universitätsklinikum Jena, Jena, Deutschland; 4 Klinik für Innere Medizin II – Hämatologie und Onkologie, Universitätsklinikum Jena, Jena, Deutschland

**Keywords:** Datenhoheit, Datenautonomie, Akutpatienten, DMP-Patienten, Diabetes-Typ-2-Patienten, Palliativpatienten, Data sovereignty, Data autonomy, Acute care patients, DMP patients, Type 2 diabetes patients, Palliative patients

## Abstract

**Hintergrund und Ziel:**

Menschen in Deutschland haben eine hohe Sensibilität bezüglich ihrer Gesundheitsdaten. So stellen sich auch in Hinblick auf die elektronische Patientenakte (ePA) Fragen zu patientenseitigem Datenhoheitsbedürfnis und Akzeptanz. Die Möglichkeit, in der ePA gespeicherte Daten selektiv weiterbehandelnden Ärzten vorzuenthalten (Opt-out), und ein Vorwissen des Patienten über die ePA könnten Einfluss auf das Datenhoheitsbedürfnis und auf die Akzeptanz der ePA haben. Das Ziel dieser explorativen Studie ist es, diese Einflüsse für die 3 Patientengruppen „Akutpatienten“, „Diabetes-Typ-2-Patienten“ und „Palliativpatienten“ zu untersuchen, da hier Unterschiede vermutet werden.

**Material und Methoden:**

Von August bis Oktober 2019 wurde eine quantitative Befragung unter 140 Patienten der genannten Gruppen durchgeführt.

**Ergebnisse:**

76,0 % der Befragten befürworten die selektive Opt-out-Möglichkeit und erklärten, dass sich ihre Bereitschaft zur Teilnahme an der ePA dadurch erhöht. Gruppenspezifisch erklärten dies 81,1 % der Akutpatienten, 80,6 % der Palliativpatienten sowie 65,6 % der Diabetes-Typ-2-Patienten. Die Unterschiede zwischen den Gruppen waren nicht signifikant. Ein generelles Vorwissen zur ePA hing mit einem höheren Datenhoheitsbedürfnis zusammen – 43,2 % derjenigen, die von der ePA-Einführung noch nie bewusst gehört hatten, würden ihre Gesundheitsdaten gelegentlich vor anderen Ärzten verbergen gegenüber 54,5 %, die von der Einführung wussten.

**Diskussion:**

Die Berücksichtigung des Datenhoheitsbedürfnisses von Patienten bei der weiteren Etablierung der ePA wird empfohlen. Die selektive Opt-out-Möglichkeit kann zur Akzeptanz beitragen. Die Kenntnisse zur ePA sollten v. a. im Arzt-Patienten-Gespräch erweitert werden, um eine informierte Entscheidung zu ermöglichen.

**Zusatzmaterial online:**

Zusätzliche Informationen sind in der Online-Version dieses Artikels (10.1007/s00103-022-03589-w) enthalten.

## Abstract (en)

**Background and aim:**

People in Germany are very sensitive about their health data. The electronic health record (ePA) also raises questions about the patient’s need for data sovereignty and acceptance. The possibility of selectively withholding data stored in the ePA from physicians who continue to treat the patient (opt-out) and the patient’s prior knowledge of the ePA could influence the need for data sovereignty and acceptance of the ePA. The aim of this explorative study is to investigate these influences for three patient groups: “acute patients,” “diabetes type 2 patients,” and “palliative patients,” as differences are expected between these groups.

**Materials and methods:**

From August to October 2019, a quantitative survey was conducted among 140 patients in the abovementioned groups.

**Results:**

Of the respondents, 76.0% supported the selective opt-out option and stated that this would increase their willingness to participate in the ePA. Specifically, 81.1% of acute care patients, 80.6% of palliative care patients, and 65.6% of type 2 diabetes patients made this statement. Differences between groups were not significant. A general prior knowledge of the ePA was related to a higher need for data sovereignty – 43.2% of those who had never heard of the ePA rollout would occasionally hide their health data from other physicians, compared with 54.5% who knew of the rollout.

**Discussion:**

Consideration of the data sovereignty needs of patients in the further establishment of the ePA is recommended. The selective opt-out option can contribute to acceptance. Knowledge of the ePA should be expanded, especially in the doctor-patient discussion, to enable an informed decision.

## Einleitung

Menschen in Deutschland besitzen eine hohe Sensibilität, wenn es um ihre persönlichen Daten geht [1]. Dies gilt insbesondere in Bezug auf persönliche Gesundheitsdaten in E‑Health-Angeboten. So wollen laut einer Umfrage des Verbandes der deutschen Informations- und Telekommunikationsbranche Bitkom e. V. aus dem Jahr 2019 61 % der Deutschen, dass die Datenhoheit bei den Patienten liegt; 59 % verlangen ein Höchstmaß an Datenschutz und Datensicherheit [2]. Für 45 % sind außerdem eine strukturierte Darstellung der eigenen Patientendaten und für 34 % ein mobiler Zugang zu diesen wichtig.

Technisch gesehen handelt es sich bei der elektronischen Patientenakte (ePA) zunächst um eine auf einem elektronischen/IT-System basierende Akte, die von einem Arzt über einen Patienten geführt wird. Sie muss den Versicherten seit 2021 standardmäßig von ihrer Krankenkasse zur Verfügung gestellt werden. Sie ist die elektronische Version der bisherigen Patientenakte und dient dem Arzt zunächst als elektronische Lösung für deren Verwaltungs- und Dokumentationsaufgaben. Darüber hinaus dient sie den verschiedenen Akteuren im Gesundheitssystem (Ärzten, Apotheken, Therapeuten etc.) als gemeinsame Plattform zum Austausch der behandlungsrelevanten Daten, wie bspw. Befunde oder auch Verordnungen.

65 % der Bundesbürger gaben 2019 an, die ePA nutzen zu wollen [2]. Die Bereitschaft zur Nutzung der ePA lag bei den 16- bis 29-Jährigen mit 74 % und bei den 30- bis 49-Jährigen mit 70 % besonders hoch. Selbst in der Altersgruppe 65 plus gaben 60 % der Befragten an, auf die ePA zugreifen zu wollen. Zu einem ähnlichen Ergebnis kam auch eine Statista-Umfrage desselben Jahres, wonach 1464 Personen zur beabsichtigten Nutzung der ePA befragt wurden: 62 % standen der Nutzung positiv gegenüber (31 % antworteten mit „Ja bestimmt“, 31 % mit „eher Ja“) und 37 % reagierten ablehnend (22 % „eher Nein“; 15 % „Nein, bestimmt nicht“; [3]).

Dem Datenhoheitsbedürfnis trug das am 01.04.2020 vom Bundeskabinett beschlossene „Patientendaten-Schutz-Gesetz“ Rechnung. Danach soll der Patient die völlige Hoheit über seine Daten erhalten und darüber entscheiden können, welche Daten in der ePA gespeichert werden oder wer auf die ePA zugreifen darf [4]. Zudem soll er seine Daten selbstständig löschen können [5].

Zum Zeitpunkt der hier durchgeführten Befragung (2019) war diese Möglichkeit, dass Patienten eine Hoheit über die Sichtbarkeit ihrer Daten haben sollen, weder sicher noch bekannt. Vor diesem Hintergrund wurde im Patientenfragebogen auf S. 4 (s. Onlinematerial) fiktiv ein entsprechendes System der Opt-out-Möglichkeit skizziert und in 4 Punkten erläutert. Dieses bildete das Ausgangsszenario für alle Fragen, welche sich auf das selektive Ausblenden von Patientendaten gegenüber anderen Ärzten bezogen.

In der vorliegenden Studie wurden akut erkrankte Patienten (A), chronisch erkrankte Patienten mit Diabetes Typ 2 (D) sowie Palliativpatienten (P) befragt, um Unterschiede in den Datenhoheitsbedürfnissen^1^ verschiedener Patientengruppen zu untersuchen, die das Gesundheitssystem unterschiedlich erleben und nutzen. Diabetes-Typ-2-Patienten wurden exemplarisch für Patienten mit einer Teilnahme an einem Disease-Management-Programm (DMP) ausgewählt – eine Gruppe mit langzeitiger und kontinuierlicher Gesundheitssystemerfahrung. Es ist ein Anliegen dieser Studie, mit einer ersten Untersuchung zukünftige Diskussionen bezüglich der Datenhoheit in Behandlungssituationen, spezifiziert nach im Sinne der Morbidität möglichst heterogenen Patientengruppen, anzustoßen, um etwaige Einflussfaktoren zur Erreichung einer höheren Akzeptanz der ePA in der Bevölkerung ermitteln zu können.

Folgende Primärfragestellungen lagen der Konzeption dieser Studie zugrunde:

1. Würde ein Konzept der generellen Archivierung von Befunden mit einer Opt-out-Möglichkeit „nicht sichtbarer Eintrag“ grundsätzlich auf Akzeptanz stoßen und die Haltung gegenüber der ePA beeinflussen?
2. Sind Unterschiede im Datenhoheitsverhalten der exemplarischen Patientengruppen akut erkrankter, chronisch erkrankter und palliativer Patienten feststellbar?
3. Nimmt das Interesse an Datenautonomie mit zunehmendem Vorwissen über die ePA zu oder ab?

## Material und Methoden

### Rekrutierung der Studienteilnehmer

Befragt wurden im Jahr 2019 über einen Zeitraum von 3 Monaten von August bis einschließlich Oktober insgesamt 150 Patienten ab einem Alter von 18 Jahren, untergliedert in die 3 Gruppen à 50 Teilnehmer:

(A) akut erkrankte Patienten,
(D): chronisch erkrankte Patienten, die an einem DMP teilnehmen, hier Diabetes-Typ-2-Patienten,
(P): onkologische Palliativpatienten, die an einer spezialisierten ambulanten Palliativversorgung (SAPV) teilnehmen.

Die Gruppen (A) und (D) wurden in einer aus 3 Ärzten bestehenden hausärztlichen Gemeinschaftspraxis rekrutiert. Sie wurden gemäß dem regulären Patientenkollektiv der Studienpraxis ausgewählt und an einem Stichtag angesprochen. Die Patientenklientel der Praxis setzt sich heterogen zusammen in Bezug auf die Alters- und Bildungsstruktur sowie die ethnische Herkunft. Die Praxis hat sowohl einen städtischen als auch einen ländlichen Einzugsbereich. Voraussetzung für die Teilnahme waren eine gute Kenntnis der deutschen Sprache und die Fähigkeit, die Fragebögen eigenständig auszufüllen. Im Befragungsjahr 2019 war diese Praxis eine reguläre, nichtdigitalisierte Hausarztpraxis, deren Patienten ihre Befunde noch in nichtdigitalisierter Form mit sich führten.

Die Gruppe (P) wurde primär durch die Palliativärzte eines Norddeutschen SAPV-Palliativstützpunktes angesprochen. Die Auswahl geeigneter Probanden erfolgte mittels subjektiver Einschätzung einer ausreichend vorhandenen Vigilanz durch die Palliativärzte.

### Befragung

Ein eigens entwickelter quantitativer Fragebogen mit 22 geschlossenen Fragen und erläuterndem Text diente zur Datenerhebung (s. Onlinematerial). Er umfasste 5 Seiten zum Selbstausfüllen/Ankreuzen durch die Patienten. Dabei wurde folgende Strukturierung vorgenommen:

- Teil A: 10 allgemeine Fragen zu Alter, Geschlecht, Bildung, Herkunft etc.,
- Teil B: 2 Fragen zum aktuellen Umgang mit ärztlichen Befunden,
- Teil C: 5 Fragen zum allgemeinen Wissensstand zum Thema „ePA“,
- Teil D: 5 spezifische Fragen zur Bewertung der „ePA“ nach Schilderung des Opt-out-Verfahrens.

Im Rahmen dieser Studie wurden die Teile A, C und D des Fragebogens einbezogen, schwerpunktmäßig wurden jedoch A zur Demographie sowie Teil D herangezogen und ausgewertet.

In Teil D des Fragebogens wurden den Teilnehmenden das selektive Opt-out-Verfahren und ihre Wahlmöglichkeit zunächst schriftlich erklärt. Beim Opt-out-Verfahren können sich die Patienten entscheiden, ob sie eher einzelne Untersuchungsergebnisse oder alle Einträge der ePA als inaktiv markieren lassen würden.

Die Einschätzung der persönlichen Einstellung erfolgte mittels Multiple-Choice-Fragen sowie 6‑stufiger Likert-Skalen. Die Likert-Stufen 1–3 wurden in der Auswertung als „eher zustimmend“, die Stufen 4–6 als „eher ablehnend“ zusammengefasst. Zusätzlich gab es die Antwortmöglichkeit „Ich weiß nicht“.

Etwaig vorhandene Kenntnisse des Unterschieds zwischen ePA und elektronischer Gesundheitskarte (eGK) sowie ihre Auswirkung auf das Datenhoheitsbedürfnis wurden in Teil C mit abgefragt.

### Statistische Methodik

Für die nominalen Items des Fragebogens wurden absolute und relative Häufigkeiten ermittelt, die grafische Darstellung erfolgte mittels gestapelter Balkendiagramme. Binäre Variablen wurden zwischen 2 Gruppen mit dem zweiseitigen „exakten Test nach Fisher“ verglichen, das Signifikanzniveau wurde bei 5 % festgelegt. Es wurde keine Korrektur für multiples Testen aufgrund des explorativen Charakters der Studie durchgeführt.

Metrische Variablen (z. B. Alter der Befragten) wurden durch Mittelwert und Standardabweichung zusammengefasst, als weitere deskriptive Maße wurden Median und Spannweite angegeben.

## Ergebnisse

Von den ursprünglich 150 angesprochenen Patienten nahmen an der Befragung entsprechend den jeweiligen Gruppen teil: (A) 50/50; (D) 50/50; (P) 40/50. Gesamt *N* = 140.

### Demographische Angaben.

58,6 % (82 von 140) der Befragungsteilnehmer waren weiblich und 41,4 % (58 von 140) männlich (Tab. 1). Das Alter der Befragten lag im Mittel bei 58,4 Jahren und im Median bei 61,0 Jahren bei einer Standardabweichung von 16,703. Dabei lag die Altersspannweite zwischen 21 und 90 Jahren. Beim Studiendesign war uns dieses relativ hohe Alter der Befragungsteilnehmer bewusst, welches sowohl der Patientenstruktur der teilnehmenden Studienpraxis als auch der Tatsache, dass ein Drittel der Befragten SAPV-Patient gewesen ist, geschuldet ist. Da diese Studie jedoch lediglich eine erste Tendenz aufzeigen und nicht als abschließend verstanden werden soll, haben wir dies bewusst in Kauf genommen. Eine Übersicht über alle erhobenen demographischen Daten des Patientenkollektivs befindet sich in Tab. 1.

**Table 1:** 

	Akutpatienten (*n* = 50)	Diabetes-Typ-2-Patienten (*n* = 50)	Palliativpatienten (*n* = 40)	Gesamt (*N* = 140)
*Alter*	51,1 ± 16,4	61,9 ± 16,9	63,6 ± 13,7	58,4 ± 16,7
*Geschlecht*				
Männlich	16 (32,0 %)	21 (42,0 %)	21 (52,5 %)	58 (41,4 %)
Weiblich	34 (68,0 %)	29 (58,0 %)	19 (47,5 %)	82 (58,6 %)
*Schulabschluss*				
Keinen Abschluss	1 (2,0 %)	1 (2,0 %)	2 (5,0 %)	4 (2,9 %)
Hauptschule	14 (28,0 %)	18 (36,0 %)	10 (25,0 %)	42 (30,0 %)
Realschule	22 (44,0 %)	19 (38,0 %)	18 (45,0 %)	59 (42,1 %)
Abitur/Fachabitur	13 (26,0 %)	11 (22,0 %)	10 (25,0 %)	34 (24,3 %)
Keine Angabe	0	1 (2,0 %)	0	1 (0,7 %)
*Höchste Berufsqualifikation*				
Keinen Abschluss/ungelernt	5 (10,0 %)	7 (14,0 %)	7 (17,5 %)	19 (13,6 %)
Lehre	35 (70,0 %)	29 (58,0 %)	22 (55,0 %)	86 (61,4 %)
Studium	9 (18,0 %)	11(22,0 %)	11 (27,5 %)	31 (22,1 %)
Keine Angabe	1 (2,0 %)	3 (6,0 %)	0	4 (2,9 %)
*Geburtsland*				
Deutschland	45 (90,0 %)	45 (90,0 %)	37 (92,5 %)	127 (90,7 %)
Anderes Land	5 (10,0 %)	5 (10,0 %)	3 (7,5 %)	13 (9,3 %)

### Bewertung der selektiven Opt-out-Möglichkeit.

**Figure 1:**
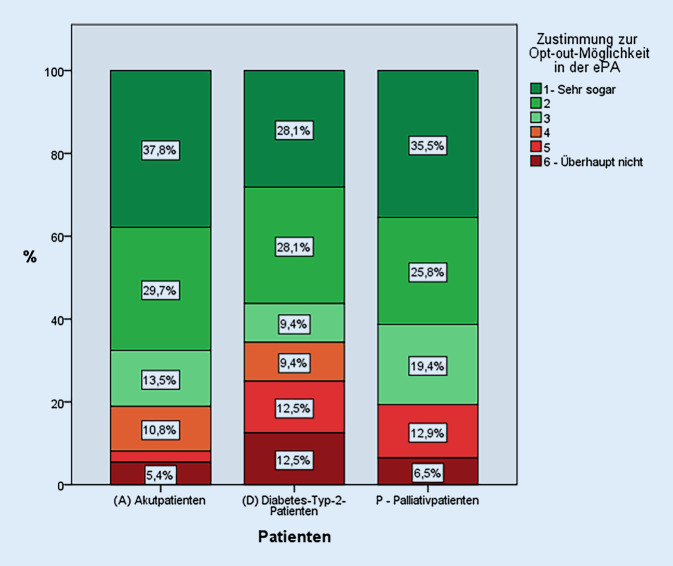

Auf die Frage Nr. 18: „Würden Sie so ein System begrüßen und Ihre Bereitschaft zur Teilnahme an einer elektronischen Patientenakte erhöhen?“, antworteten 76,0 % (76 von 100 antwortenden Patienten) mit den Antworten 1–3 auf der Likert-Skala „eher zustimmend“. Getrennt betrachtet taten dies 81,1 % (30 von 37) der (A) akut erkrankten Patienten, 65,6 % (21 von 32) der (D) Diabetes-Typ-2-Patienten und 80,6 % (25 von 31) der (P) Palliativpatienten (Abb. 1). Dabei sind die Unterschiede in der Zustimmung zwischen den Gruppen nicht signifikant (*p* = 0,249).

Auch lässt sich bei Frage 18, nach Geschlechtern getrennt betrachtet, kein signifikanter Unterschied zwischen „eher zustimmend“ oder 4–6 „eher ablehnend“ feststellen (*p* = 1,000, exakter Test nach Fisher). Der Anteil an Zustimmung mit 76,4 % (42 von 55) bei den weiblichen Befragten ist ähnlich dem der männlichen Befragten mit 75,6 % (34 von 45).

### Generelle Bewertung von Datenhoheit durch Patienten nach Gruppe – ePA-unabhängig.

Bei Frage 19: „Betrachten Sie ihre Gesundheitsdaten als so schützenswert, dass Sie diese gelegentlich auch vor Zugriffen anderer Ärzte schützen möchten?“, reagierten 54,3 % der antwortenden Palliativpatienten (19 von 35; P) zustimmend. Entsprechend sind 45,7 % (16 von 35) generell offen gegenüber einer Datenweitergabe. Innerhalb der Gruppe der Diabetes-Typ-2-Patienten (D) ist das Verhältnis mit jeweils 50 %/50 % (20 von 40) genau ausgeglichen (*p* = 0,914). Über alle Gruppen betrachtet lag die Offenheit der Weitergabe der Gesundheitsdaten an weitere Behandler bei 48,7 % (56 von 115).

### Auswirkung von Vorwissen über ePA zur Datenfreigabebereitschaft.

Unter den 37 Befragten, die in Frage 13: „Haben Sie schon einmal von der Einführung der ‚elektronischen Patientenakte‘ (ePA) gehört?“, angaben, dass sie bis zu dieser Umfrage zuvor noch nie bewusst von der Einführung der ePA gehört hatten, hielten nur 43,2 % (16 von 37) ihre Gesundheitsdaten für schützenswert, sodass sie fallweise bestimmten Ärzten keinen Einblick gewähren würden; 56,8 % (21 von 37) dieser Gruppe hingegen sahen die Einsichtnahme anderer Ärzte in ihre Gesundheitsdaten unkritisch. Unter den Befragten, die bereits von der geplanten Einführung der ePA gehört hatten, kehrte sich dieses Verhältnis jedoch nahezu um: Dann erachteten 54,5 % (42 von 77) der Befragten ihre Gesundheitsdaten als so schützenswert, dass diese selektiv einigen Ärzten nicht zugänglich gemacht werden sollen. 45,5 % (35 von 77) dieser Gruppe hatten mit der Einsichtnahme keine Probleme (*p* = 0,318).

Noch deutlicher wurde es, wenn die Befragten angaben, den Unterschied zwischen elektronischer Gesundheitskarte (eGK) und ePA zu kennen. Von diesen erachteten 60,7 % (17 von 28) ihre Gesundheitsdaten als so schützenswert, dass sie sie selektiv bestimmten Ärzten vorenthalten würden, wohingegen lediglich 39,3 % (11 von 28) dieser Befragten die Einsichtnahme weiterer Ärzte in ihre Gesundheitsdaten unkritisch sahen. Unter denjenigen Befragten, die angaben, den Unterschied zwischen eGK und ePA nicht zu kennen, verhielt es sich nahezu umgekehrt, wenngleich auch nicht signifikant (*p* = 0,518): 48,2 % (41 von 85) erachteten ihre Gesundheitsdaten als selektiv schützenswert gegenüber anderen Ärzten, 51,8 % (44 von 85) nicht.

Unter jenen Befragten, die wussten, dass die ePA in Deutschland eingeführt werden soll (Frage 15: „Wissen Sie, ob die elektronische Patientenakte auch in Deutschland bereits eingeführt wurde oder wird?“), erachteten 66,0 % (31 von 47) ihre persönlichen Gesundheitsdaten als so schützenswert, dass sie diese selektiv bestimmten Ärzten vorenthalten möchten. Von den Befragten, die angaben, von der geplanten ePA-Einführung in Deutschland nichts zu wissen, waren es hingegen nur 39,1 % (25 von 64), die ihre Gesundheitsdaten in bestimmten Situationen anderen Ärzten vorenthalten würden, gegenüber 60,9 % (39 von 64), die ihre Daten nicht vorenthalten würden. Der Unterschied zu der Gruppe der Befragten, die angaben, von der Einführung zu wissen, war signifikant (*p* = 0,007).

Wissen die Befragten laut Frage 16: „Ist Ihnen bekannt, dass außer Ihrem Arzt auch Sie selbst bei einer elektronischen Patientenakte über das Internet mittels Computer oder Smartphone jederzeit Einblick in Ihre Patientenakte mit all den dort hinterlegten Befunden und Diagnosen haben können?“, dass sie in der geplanten ePA neben den Ärzten und sonstigen Einsichtsberechtigten online auch selbst Einblick in ihre Gesundheitsdaten nehmen können, liegt der Wunsch nach selektivem Datenvorbehalt bei 45,0 % (9 von 20) gegenüber 55,0 % (11 von 20), die ihre Daten nicht schützen würden. Der Unterschied ist mit *p* = 0,629 nicht signifikant.

## Diskussion

### Akzeptanz der Opt-out-Möglichkeit und Einfluss auf die Einstellung zur ePA

Unsere Befragung zeigte, dass eine Mehrheit von 76,0 % (76 von 100 antwortenden Patienten) gegenüber der Möglichkeit, die ein selektives Ausblenden von Gesundheitsdaten bzw. Befunden innerhalb der ePA bietet, positiv eingestellt ist. Hierbei ist die Zustimmung mit 81,1 % (30 von 37) unter den akut Erkrankten (A) am größten, gefolgt von der Gruppe der Palliativpatienten (P) mit 80,6 % (25 von 31) sowie 65,6 % (21 von 32) unter den Diabetes-Typ-2-Patienten (D). Hierbei handelt es sich jedoch um keinen signifikanten Unterschied (*p* = 1,000, exakter Test nach Fisher).

Zumindest für die Gruppe der Diabetes-Typ-2-Patienten (D) haben wir dieses Ergebnis so nicht erwartet, da wir davon ausgingen, dass das Datenhoheitsbedürfnis von Diabetespatienten größer als in anderen Patientengruppen sein müsste [7]. Zu dieser Annahme gelangten wir, weil Diabetes-Patienten für eine geringere Therapietreue (Essverhalten, Lebensstil) bekannt sind und jährlich im Durchschnitt ca. 3–4 verschiedene Ärzte aufsuchen [7]. Es läge also im Interesse der Patienten, Daten zur nicht eingehaltenen Therapie vor weiteren Ärzten auszublenden. Dass dem jedoch nicht zwingend so sein muss, belegt auch Kulzer [4, 8]. Eine Untersuchung von amerikanischen Diabetespatienten aus dem Jahr 2019 hatte nämlich gezeigt, dass ältere Personen mit einer in der Regel größeren Gesundheitssystemerfahrung eine dort implementierte ePA sogar leicht häufiger nutzten als jüngere [9].

So stellten Kulzer et al. bei einer Onlinebefragung von 3427 Patienten mit Diabetes fest, dass es unter diesen zwar mit 83 % eine positive bis sehr positive Einstellung gegenüber der Digitalisierung in der Medizin – hier primär der diabetesbezogenen Medizin – gibt, die Gefahr des Missbrauchs von Patientendaten aber nur von 32 % dieser Gruppe gesehen wird [8].

Dass die Palliativpatienten ihre Daten mit 54,3 % (19 von 35) für etwas schützenswerter hielten als die anderen Gruppen, mag darin begründet liegen, dass diese sie sich in ihrer Palliativsituation ohne Perspektive auf Heilung befinden [10]. Um diese Annahme zu untermauern, bedürfte es hier jedoch einer gesonderten Untersuchung, da unser Studiendesign Abfragen nach den dahinterliegenden Begründungen der Befragten nicht vorgesehen hat. Gleiches würde für ein detaillierteres Abklären der Motivationen weiterer Patientengruppen sprechen. Dabei müsste der zu erwartende Erkenntnisnutzen in Anbetracht des nicht geringen Aufwandes, insbesondere bei einem größeren und erweiterten Panel, zuvor noch einmal bedacht werden.

### Einfluss von Vorwissen zur ePA auf das Interesse an Datenautonomie

Es konnte beobachtet werden, dass Befragte mit mehr Kenntnis der ePA – über alle 3 Patientengruppen hinweg – weniger bereit waren, eigene Gesundheitsdaten in der ePA weiteren Ärzten zugänglich zu machen. Wenn die Befragten allerdings wussten, dass auch sie selbst Einblick in ihre ePA nehmen können, waren mit 55,0 % (9 von 20) etwas mehr Personen zur Datenfreigabe bereit. Dies lässt die Überlegung zu, dass in diesem Fall die ePA möglicherweise weniger als „Blackbox“ angesehen wird, was das Nutzervertrauen steigern könnte.

Im Jahr 2020 gaben laut Wallenfels 64,0 % von 868 befragten Befürwortern der ePA an, dass die Datenhoheit über die Daten beim Patienten liegen sollte [11]. Zudem wurden Datenschutz und Datensicherheit von 63,0 % als die zweitwichtigste Voraussetzung für die Nutzung der ePA benannt [11]. Dies stützt nach unserer Ansicht unsere zuvor beschriebenen Beobachtungen, dass Datenhoheit als ein Akt der Selbstwirksamkeit bewusst und situationsangepasst von den Patienten wahrgenommen wird, wenn ihnen differenzierte Informationen diesbezüglich zur Verfügung stehen.

Die vorliegenden Ergebnisse müssen im Kontext des Versorgungsalltags betrachtet werden: Grundlage einer erfolgreichen Arzt-Patienten-Beziehung ist Vertrauen, nicht zuletzt auch im Sinne der partizipativen Entscheidungsfindung (Shared Decision Making), welche Basis für eine gelingende informierte Einwilligung der Patienten (Informed Consent) ist [12]. So käme es der Einschätzung der Autoren nach auf das Gespür des jeweiligen Arztes – insbesondere im Setting der Niedergelassenen – an, das jeweils unterschiedliche Vorwissen des Patienten zur ePA und dessen Validität als auch die Grundhaltung dieser gegenüber der ePA zu eruieren. Eine Aufgabe, die im oft streng durchgetakteten Behandlungsalltag allenfalls von langjährig betreuenden Hausärzten geleistet werden kann, da hier i. d. R. von einer soliden Arzt-Patienten-Vertrauensbasis ausgegangen werden kann.

Dem entgegen steht jedoch, dass auch bei den niedergelassenen Ärzten in Deutschland kein einheitliches Akzeptanzbild gegenüber der ePA besteht. Der Enthusiasmus wird durch hohe Gebühren und die häufigen technischen Probleme beim Anschluss an die Gematik-IT-Struktur gemindert [13]. Die Motivation, den Patienten die ePA näherzubringen, wird dadurch eventuell negativ beeinflusst.

Dass seitens vieler Patienten ein Interesse an den Themen ePA, Datenschutz und Datenhoheit besteht, lässt sich im Rahmen der vorliegenden Studie vielleicht auch an der Beobachtung der Praxismitarbeiter erkennen, dass Patienten während des Ausfüllens ihres Fragebogens im Wartezimmer häufig eine interessierte Diskussion mit anderen wartenden Patienten begannen. Auch berichteten die beteiligten Ärzte, dass manche Patienten im anschließenden Behandlungsgespräch gemeint hatten, sich zuvor noch nie Gedanken über die Fragen ihrer Datenhoheit gemacht zu haben. Diese Beobachtungen können als Anregung gesehen werden, zukünftig näher zu untersuchen, inwieweit ein patientenseitiges Reflektieren über diese Fragestellungen generell vorhanden ist und ob es hier weitergehender, öffentlicher Informationskonzepte bedarf. In einer Zeit, in der die meisten Menschen bei der Installation neuer Software oder Apps die Datenschutz- und Datenverwertungserklärung ungelesen akzeptieren, wird insbesondere darauf zu achten sein, dass die Datenhoheitskompetenz weiterhin bei den Patienten vorhanden bleibt oder ausgebaut wird [14].

Auch wenn es darum geht, digital vorhandene Patientendaten – wenngleich auch anonymisiert – der Medizin- und Gesundheitsindustrie zur Verfügung zu stellen, wird ein patientenzentriertes Datenhoheitskonzept der ePA als notwendig zu erachten sein. Dies muss insbesondere auch im Kontext der EU-weit gültigen Datenschutzgrundverordnung (DSGVO) ein jederzeit anwendbares Rechtsgut der nachträglichen Nutzungseinschränkung durch die Patienten sein [15].

### Limitationen

Da es sich hier um eine kleine explorative Studie handelt, ist zu überlegen, diese Untersuchung in einem größeren und erweiterten Setting, idealerweise auch unter Einbeziehung jüngerer sowie auch stationärer Patienten, erneut durchzuführen. Ferner wären in einem weiteren Schritt auch Experteninterviews sowohl unter Behandlern als auch unter sonstigen Akteuren des Gesundheitssystems zu empfehlen, um auch deren Voten in etwaige Überlegungen zur Implementierung dieser Opt-out-Option in die Architektur der ePA einzubeziehen.

## Fazit

Die Interpretation unserer Daten legt nahe, dass ein Eingehen auf das Datenhoheitsbedürfnis von Patienten mittels der Möglichkeit der selektiven Ausblendung von Gesundheitsdaten in der ePA (Opt-out) eine Verbreiterung der Akzeptanzbasis für die ePA unterstützen würde. Generell sollte der Datenhoheit der Patienten bei der Entwicklung von E‑Health-Komponenten eine zentrale Rolle zukommen [14].

Spezifisches Vorwissen über die ePA als auch die Zugehörigkeit zu unterschiedlichen Patientengruppen veranlasst die Patienten zu einem differenzierten Umgang mit ihren Daten im Sinne der Datenhoheit. Die von uns befragte Opt-out-Möglichkeit, bei der der Datensatz und somit die Datenkonsistenz vollständig erhalten bleibt, da Einträge nicht gelöscht, sondern lediglich unsichtbar gesetzt werden können, stellt ein entsprechendes Instrument für die Patienten dar. Im Rahmen des in der „ePA 2.0“ vorgesehenen, verfeinerten Berechtigungsmanagements, welches für die ePA in Deutschland zu Jahresbeginn 2022 eingeführt wurde, wird dem in dieser Studie skizzierten Opt-out-Ansatz Rechnung getragen [16].

## Supplementary Information





## Footnotes

1 Anmerkung: Der Fokus dieser Untersuchung liegt ausschließlich auf dem für die Patienten primären Datenhoheitsbedürfnis gegenüber anderen Ärzten und Behandlern in der Weiterbehandlungssituation und nicht auf der inzwischen ebenfalls diskutierten, anonymisierten Datenweitergabe von Patientendaten aus der ePA zu Forschungszwecken [6].
