# Supplementary material for: Patient group-specific data sovereignty needs and acceptance of electronic health records
Source: Bundesgesundheitsblatt Gesundheitsforschung Gesundheitsschutz. 2022 Sep 23;65(11):1197–203. [Article in German] doi: 10.1007/s00103-022-03589-w (PMC9636088; doi:10.1007/s00103-022-03589-w)
Supplement: Supplementary file 1 [file 103_2022_3589_MOESM1_ESM.pdf]

# F r a g e b o g e n z u r ‚ e l e k t r o n i s c h e n P a t i e n t e n a k t e ’

Hameln, TT.MM.2019

Sehr geehrte Damen und Herren,

gemeinsam mit der Universitätsklinik Jena führe ich eine Studie zur sogenannten *bundeseinheitlichen ‚elektronischen Patientenakte (ePA)’* durch. Das Ziel der Befragung ist es heraus zu finden, welche Informationen in der Bevölkerung zu diesem Thema vorhanden sind und welche Art des Umgangs sich Patienten mit ihren persönlichen Gesundheitsdaten wünschen.

**Diese Befragung wird bewusst anonym durchgeführt! Eine eindeutige Zuordnung zu Ihnen als Person, auch im Nachhinein erfolgt nicht! Dies wäre für diese Art der Befragung auch überhaupt nicht notwendig. Mit dem Ausfüllen dieses Fragebogens geben Sie Ihre Einwilligung zur Auswertung ihre anonymen Daten.**

Beziehen Sie sich bitte bei Fragen zu Ärzten auf Ihre Erfahrung mit Ärzten generell, und nicht ausschließlich auf diese Arztpraxis, in der Sie diesen Fragebogen erhalten haben!

**>> Bitte füllen Sie den Fragebogen in der Reihenfolge von Anfang zum Ende aus und springen Sie bitte nicht mehr zu vorgehenden Fragen zurück.**

**>> Sofern nicht extra auf die Möglichkeit der Mehrfachnennung hingewiesen wird, kreuzen Sie bitte jeweils nur eine Antwortmöglichkeit an.**

Zur besseren Lesbarkeit wurde die männliche Form gewählt ohne jedoch ein Geschlecht diskriminieren zu wollen!

Ich danke Ihnen schon jetzt recht herzlich für Ihre Teilnahme,

Dr. Marc Baron v. Osthoff

**A. Allgemeine Fragen**

01 Alter: _____

02 Geschlecht:

- o 1 weiblich
- o 2 männlich
- o 3 divers

03 Ihr höchster Schulabschluss

- o 1 keinen Abschluss
- o 2 Hauptschule
- o 3 Realschule
- o 4 Abitur / Fachabitur

04 Ihre höchste Berufsqualifikation

- o 1 keinen Abschluss / ungelernt
- o 2 Lehre
- o 3 Studium

05 In welchem Land sind Sie geboren?

o 1 Deutschland      o 2 Anderes Land: ________________________________________

*[Bitte in DRUCKBUCHSTABEN.]*

06 Wenn Sie nicht in Deutschland geboren wurden, wie lange leben Sie schon in Deutschland? ______ Jahre

07 Muss Ihnen jemand diesen Fragebogen aus dem Deutschen in eine andere Sprache übersetzen?

o 1 Ja      o 2 Nein

08 Gehen Sie mindestens einmal pro Woche ins Internet?

Ja *[Mehrfachnennung möglich.]*

- o 1 Per Computer
- o 2 Per Smartphone
- o 3 Mobil per Laptop / Tablett
- o 4 Nein

09 Besitzen Sie *[Mehrfachnennung möglich.]*

- o 1 eine Smartwatch (Apple Watch, Samsung Gear, etc.)
- o 2 Fitness-Tracker (Fitbit, POLAR, Garmin etc.)
- o 3 keines der oben genannten

10 Informieren Sie sich vor einem Arztbesuch im Internet über Ihre Krankheitsanzeichen?

o 1 Ja      o 2 Nein

**B. Ihre Befunde und Unterlagen beim Arzt heutzutage**

11 Führen Sie ihre eigene Befundsammlung und fragen Sie gezielt nach Kopien Ihrer Facharztbriefe, Blutuntersuchungen, Röntgenbefunde etc.?

Ja *[Mehrfachnennung möglich.]*

- o 1 Um selbst Vergleiche z.B. von Blutbefunden von aufeinander folgenden Untersuchungen anzustellen.
- o 2 Zur Weitergabe an parallel behandelnde Ärzte.
- o 3 Um alle Krankheitsdaten für spätere Behandlungen, Kur- oder Rentenanträge griffbereit zu haben.
- o 4 Nein

12 Wenn einem Arzt zuvor alle Befunde offengelegt werden, wie wirkt sich dies Ihrer Meinung nach auf den Termin bei Ihrem Arzt aus?

- o 1 Er ist informierter und konzentriert sich mehr auf das, was ich ihm berichte.
- o 2 Er sich bereits eine Meinung gebildet und hört mir daher nicht mehr so genau zu.
- o 3 Er wird sich dennoch zuvor immer erst eine eigene Meinung bilden, bevor er die Vorbefunde liest.

**C. Allgemeine Fragen über Ihr Wissen zur ‚elektronischen Patientenakte'**

13 Haben Sie schon einmal von der Einführung der ‚elektronischen Patientenakte' (ePA) gehört?

o 1 Ja    o 2 Nein

14 Kennen Sie den Unterschied zwischen ‚elektronischer Gesundheitskarte' (eGK) und ‚elektronischer Patientenakte' (ePA)?

o 1 Ja    o 2 Nein

15 Wissen Sie, ob die elektronische Patientenakte auch in Deutschland bereits eingeführt wurde oder wird?

- o 1 Sie ist bereits eingeführt.
- o 2 Sie soll eingeführt werden.
- o 3 In Deutschland wird sie nicht eingeführt.
- o 9 Ich habe keine Ahnung.

16 Ist Ihnen bekannt, dass außer Ihrem Arzt auch Sie selbst bei einer elektronischen Patientenakte über das Internet mittels Computer oder Smartphone jederzeit Einblick in Ihre Patientenakte mit all den dort hinterlegten Befunden und Diagnosen haben können?

o 1 Ja    o 2 Nein

17 Welche Aussage trifft am ehesten auf Sie zu: Wenn ich über das Internet selbst Einblick auf all meine Gesundheitsdaten und Befunde hätte, würde ich mich..

- o 1 informierter fühlen.
- o 2 überfordert fühlen und überlasse das lieber den Ärzten.
- o 9 Das kann ich nicht einschätzen.

**D. Kurz noch ein paar besondere Fragen zur ‚elektronischen Patientenakte'**

In Deutschland ist die Einführung einer ‚elektronischen Patientenakte - ePA' – geplant. Hierbei werden die Untersuchungsergebnisse und Befunde der einzelnen Ärzte, zum Beispiel Diagnosen, Röntgenbilder oder Laborbefunde, aber auch Informationen wie beispielsweise verordnete Medikamente zentral gesichert.

Damit der Arzt diese Daten einsehen, oder auch neue Befunde speichern kann, müssen sowohl die ‚elektronische Gesundheitskarte - eGK' (das ist Ihre Versichertenkarte von der Krankenkasse) als auch der ‚elektronische Praxisausweis' Ihres Arztes in ein entsprechendes Lesegerät eingeschoben sein.

Grundsätzlich kann Ihr Arzt dann alle zuvor gespeicherten Befunde und Diagnosen, auch die der anderen Ärzte, einsehen.

Einige Menschen haben dagegen Bedenken und würden am liebsten von ihrem Recht der „Datenhoheit" Gebrauch machen, indem sie diese neuen Daten gar nicht erst in der elektronischen Patientenakte speichern lassen würden, damit spätere Ärzte diese Untersuchungsergebnisse nicht einsehen können.

Nun kann es aber für einen behandelnden Arzt hilfreich und oft auch wichtig sein, dass er die Ergebnisse und Befunde anderer Ärzte kennt.

Aus diesem Anlass wird hier ein **neues System vorgeschlagen**, um beiden Seiten gerecht zu werden – dem Recht des Patienten über die Hoheit seiner Daten sowie das Interesse des Arztes, ihm alle notwendigen Informationen für eine gute Arbeit zur Verfügung zu stellen:

1. Zunächst werden alle Untersuchungsergebnisse, Befunde etc. **zwingend** in der elektronischen Gesundheitsakte gespeichert.

2. Anschließend können die Patienten **sofort entscheiden**, ob diese neuen Befunde später für weitere Ärzte sichtbar sein sollen, oder nicht.

3. Entscheidet ein Patient oder Patientin, dass ein Befund für spätere Ärzte nicht sichtbar sein soll, können die folgenden Ärzte die Befunde nicht einsehen.

   *Es wird den folgenden Ärzten dann aber **ein Hinweis angezeigt**, dass hier ein gesperrter Befund vorhanden ist, sowie in welchem medizinischem Fachbereich dieser Befund an welchem Datum erhoben wurde; zum Beispiel: „1 gesperrter Eintrag Orthopädie vom <Datum>!"*

4. Der Arzt kann dann abwägen, ob ein Vorbefund aus jenem Fachbereich für seine weitere Behandlung hilfreich und wichtig wäre und dann **den Patienten bitten**, ihm vielleicht doch Einblick in die Vorbefunde zu geben.

   Dabei kann der Arzt evtl. Vorbehalte und Bedenken mit dem Patienten besprechen, und diese im gegebenenfalls ausräumen.

18 Würden Sie so ein System begrüßen und Ihre Bereitschaft zur Teilnahme an einer elektronischen Patientenakte erhöhen? *[Bitte geben Sie Ihre Zustimmung von 1-6 an oder ‚Ich weiß nicht']*

| O | O | O | O | O | O | O |
|---|---|---|---|---|---|---|
| 1 | 2 | 3 | 4 | 5 | 6 | 9 |
| Sehr sogar | | | | | Überhaupt nicht | Ich weiß nicht |

19 Betrachten Sie ihre Gesundheitsdaten als so schützenswert, dass Sie diese gelegentlich auch vor Zugriffen anderer Ärzte schützen möchten?

- o 1 Ja, abhängig von der Fachrichtung des Arztes
- o 2 Ja, abhängig von der Person des Arztes
- o 3 Ja, aus beiden zuvor genannten Gründen
- o 4 Nein
- o 9 Ich weiß nicht

20 Für wie wahrscheinlich halten Sie es, dass Patienten zunächst Einträge und Befunde für andere Ärzte sperren, sich dann aber durch Argumente des Arztes im Einzelfall doch davon überzeugen lassen diese Daten freizugeben? *[Bitte geben Sie Ihre Zustimmung von 1-6 an oder ‚Ich weiß nicht']*

| O | O | O | O | O | O | O |
|---|---|---|---|---|---|---|
| 1 | 2 | 3 | 4 | 5 | 6 | 9 |
| Sehr wahrscheinlich | | | | | Sehr unwahrscheinlich | Ich weiß nicht |

21 Hätten Sie Verständnis dafür, wenn Patienten auch Nachteile bei einer Behandlung in Kauf nehmen müssten, weil dies gewisse Daten dem behandelnden Arzt nicht zeigen wollen?
(Nachteile könnten zum Beispiel die Ablehnung einer weitergehenden Behandlung durch den Arzt, weil Patienten das Ergebnis eines HIV-Tests nicht offen legen wollen?)
*[Bitte geben Sie Ihre Zustimmung von 1-6 an oder ‚Ich weiß nicht']*

| O | O | O | O | O | O | O |
|---|---|---|---|---|---|---|
| 1 | 2 | 3 | 4 | 5 | 6 | 9 |
| Sehr viel Verständnis | | | | | Überhaupt kein Verständnis | Ich weiß nicht |

22 Sollte generell vorgeschrieben werden, dass in einer akut lebensbedrohlichen Situation (Notfall) der Arzt mit einem Notfallcode auf alle Einträge, und somit auch auf gesperrte Daten Zugriff hätte?
*[Bitte geben Sie Ihre Zustimmung von 1-6 an oder ‚Ich weiß nicht']*

| O | O | O | O | O | O | O |
|---|---|---|---|---|---|---|
| 1 | 2 | 3 | 4 | 5 | 6 | 9 |
| Ja, auf jeden Fall | | | | | Nein, auf gar keinen Fall | Ich weiß nicht |
